# Clinical and laboratory findings of rhabdomyolysis in opioid overdose patients in the intensive care unit of a poisoning center in 2014 in Iran

**DOI:** 10.4178/epih.e2017050

**Published:** 2017-11-08

**Authors:** Khoshideh Babak, Arefi Mohammad, Ghorbani Mazaher, Akbarpour Samaneh, Taghizadeh Fatemeh

**Affiliations:** 1 Baharloo Hospital, Tehran University of Medical Sciences, Tehran, Iran; 2 Department of Forensic Medicine, School of Medicine, Tehran University of Medical Sciences, Tehran, Iran; 3 Department of Epidemiology and Biostatistics, School of Public Health, Tehran University of Medical Sciences, Tehran, Iran

**Keywords:** Laboratory findings, Toxicity, Opioid, Rhabdomyolysis

## Abstract

**OBJECTIVES:**

The aim of this study was to investigate the clinical and demographic characteristics and some laboratory findings of hospitalized patients with acute opioid toxicity and rhabdomyolysis.

**METHODS:**

This cross-sectional study investigated 354 patients hospitalized at Baharloo Hospital in Tehran in 2014 with acute illicit drug toxicity. Data were collected using an investigator-made checklist. The collected data (such as mortality rate, demographic data, and renal function tests, as well as serum biochemical findings) were analyzed by descriptive statistics and the chi-square test.

**RESULTS:**

A total of 354 patients were admitted to the hospital in 2014 with acute illicit drug toxicity, including 291 males and 63 females. The total number of patients with rhabdomyolysis was 76 (21.5% of the total), of whom 69 (90.8%) were male and 7 (9.2%) were female. Most cases of rhabdomyolysis were associated with methadone abuse, followed by opium abuse. Rhabdomyolysis was most common in those 20-29 and 30-39 years old, with methadone and opium the most commonly abused illicit drugs. The mean blood urea level was 3.8±1.0 mg/dL, and the mean serum potassium and sodium levels were 3.8±0.3 mg/dL and 140.4±4.0 mg/dL, respectively. Five patients, all of whom were male, passed away due to severe renal failure (6.5%).

**CONCLUSIONS:**

Toxicity caused by opioids is associated with clinical complications and laboratory disorders, such as electrolyte disorders, which can lead to lethal or life-threatening results in some cases. Abnormal laboratory test findings should be identified in patients with opioid toxicity in order to initiate efficient treatment.

## INTRODUCTION

Rhabdomyolysis syndrome is defined by the degradation of muscle cells and the dissemination of muscle cellular compounds throughout the bloodstream. This disorder may result in the release of muscle cell compounds into the extracellular and circulatory fluid, including potassium, lactate dehydrogenase, uric acid, and myoglobin. One of the key elements released is myoglobin [1]. There are several reasons for rhabdomyolysis, which are broadly divided into 3 categories: impacted muscle contraction (for example, crushing syndrome or prolonged immobilization), extreme physical activities (in people without hyperthermia or sympathetic nervous system disorders) and non-exertional and non-traumatic causes (drugs or toxins, infection, or electrolyte disorders) [2,3].

Rhabdomyolysis can easily be detected after severe and critical events such as muscle contraction or crash syndrome, coma or seizure, surgical trauma, and extreme physical activities, but in some other cases, the identification of rhabdomyolysis requires a careful physical examination, such as a history and laboratory evaluation [2-4]. Several prescription drugs use, as well as drug abuse, can cause rhabdomyolysis [2]. In addition to alcohol, other drugs, including heroin, cocaine, amphetamine, methadone, tramadol, and LSD, play significant roles in the development of rhabdomyolysis.

The drugs that most commonly cause rhabdomyolysis are as follows: antipsychotics, statins, selective serotonin inhibitors, zidovudine, colchicine, lithium, and antihistamines [3]. Acute intoxication with opioids and other drugs of abuse is a routine daily occurrence in emergency departments and intensive care units (ICUs), and many deaths occur due to overdose. Mortality due to overdose among users of opioids and other similar compounds is very high. Moreover, acute poisoning with these drugs not only leads to severe and potentially fatal complications during admission, but also has permanent sequelae. Rhabdomyolysis can occur after opiate overdoses, but is often not diagnosed. Opium is rapidly metabolized in the liver, which reduces the duration of its activity, even after an overdose.

Due to the debilitating consequences of rhabdomyolysis after opioid overdoses, such as acute renal damage and failure, an accurate evaluation, proper diagnostic procedures, and suitable therapeutic methods can help reduce the mortality rate among the affected patients.

Moreover, insufficient studies have been conducted in this field, so it should be an area of special focus in epidemiological and public health research.

The aim of this study was to investigate the demographic and clinical characteristics and some laboratory findings of patients with acute opioid intoxication admitted to Baharloo Hospital who developed rhabdomyolysis. We hope that the results of this study will contribute to other analytical and empirical studies, with the goal of finding appropriate protocols for detecting this condition through clinical and laboratory examinations.

## MATERIALS AND METHODS

This cross-sectional study was carried out at Baharloo Hospital in Tehran, among patients who were hospitalized in the ICU in 2014 due to poisoning with methadone, tramadol, opium, heroin, and other opioid drugs. They had creatine phosphokinase (CPK) levels of over 1,500 IU/L, without trauma, sepsis, or other drug poisoning.

The diagnosis of rhabdomyolysis was based on dark urine without other symptoms or an acute neuromuscular illness together with an acute elevation in serum creatine kinase (CK). The diagnosis of rhabdomyolysis was defined as 5 times the upper normal limit of CK (> 5,000 IU/L).

Information was collected from patient histories, physical examinations, written nursing reports, and patient laboratory exams. Data on the patients’ age, type of drug, urine culture results, and tests of CPK and lactate dehydrogenase levels and of kidney function were extracted from patients’ records.

In clinical examinations, attention was paid to issues such as fever, stiffness, muscle swelling, and urine discoloration, and attempts were made to exclude other causes of elevated CPK (such as heart attack and brain injury).

The design and method of this study were approved by the institutional review board of Tehran University of Medical Sciences, and all participants provided written informed consent.

The data of this study were entered in SPSS version 22 (IBM Corp., Armonk, NY, USA) in order to analyze the linkage between quantitative means in qualitative groups when a normal distribution was present, using the t-test and analysis of variance, and to determine the relationship between qualitative variables using the chi-square test.

## RESULTS

The total number of admitted patients in 2014 was 354, of whom 291 (82.2%) were male and 63 (17.8%) female. The age of the youngest patient was 14 and that of the oldest was 83. The mean age was 37.69± 5.87. The most common age groups was 20-29 and 30-39 years old. The most commonly used drugs were tramadol (35.9%), opium (33.0%), and methadone (28.5%), but in patients with rhabdomyolysis, the most commonly used drug was methadone (Table 1).

The total number of patients with rhabdomyolysis was 76 (21.5%); 69 patients (90.8%) were male and 7 (9.2%) were female. The number of patients with discolored urine was 18 (23.6%), while 35 patients (46.0%) had clear urine. As illustrated in Table 2, the most commonly reported drug of abuse in cases of nephrotoxicosis was methadone, followed by opium. Five patients (6.5%) all males, passed away during the study. The average age among males was 35.4 ± 13.8, and 34.0 ± 18.4 among females.

The average values of laboratory variables are shown in Table 3.

## DISCUSSION

Rhabdomyolysis, which can lead to acute renal failure and death, is one of the most common complications of acute poisoning due to alcohol, narcotics, and psychotropic drugs. Therefore, in order to reduce the complications and mortality associated with this phenomenon, early diagnosis and clinical suspicion are important; awareness of symptoms and early treatment are especially significant. Rhabdomyolysis has been reported as a clinical problem in previous studies, but recent studies have shown a higher incidence of this disorder [5]. It is one of the main causes of acute renal failure [3,6]. However, insufficient comprehensive studies have been conducted in this field. In many studies, alcohol abuse has been recognized as a main cause of rhabdomyolysis [7,8]; however, other studies have also examined the relationship between rhabdomyolysis and drug and alcohol abuse. For instance, in Iran, the reviews of Mousavi et al. [9] and Talaie et al. [7] of the etiology of rhabdomyolysis have reported opioid overdose to be a significant factor. Moreover, in the studies of Koffler et al. [10], Gabow et al. [11], and Taheri et al. [12], alcohol and opium abuse have been reported to be the main causes of rhabdomyolysis. A high incidence of rhabdomyolysis was found in the patients in their studies (21.4%). In other studies, the incidence has ranged from 7.0% in the study of Lie et al. [13] to 79.0% in that of Talaie et al. [7]. In the present study, the most frequent age range was 21-30 years old (33.3%), and a strong male predominance was found (82.2%), which is similar to previous studies. The most commonly reported narcotic drug reported in this study was tramadol (35.9%), unlike the findings of the study conducted by Frenk et al. [14], in which the most opioid type was opium (60.4%). This may have been due to an increase in the prevalence of tramadol use among young people and changes in drug abuse trends in recent years. Islambulchilar et al. [15], in a study of 1,342 poisoned patients, came to the conclusion that 5.4% of all hospital admissions were intoxication patients. In the aforementioned study, opioid abuse was the most common cause of poisoning (60.8%), followed by benzodiazepines (40.3%) and antidepressants (32.0%). Talaie et al. [7] found that opium poisoning was the most common cause of rhabdomyolysis (23.3%), followed by poisoning with benzodiazepines, phenobarbital, propranolol, aluminum phosphide, alcohol, and co-poisoning.

Studies on drug poisoning have often focused on demographic and clinical manifestations. Laboratory findings have only been carefully evaluated in a few cases of narcotic poisoning in humans, although laboratory abnormalities caused by narcotics have been studied in laboratory animals.

The mean urea level of patients with rhabdomyolysis was measured at 3.8 ± 1.0 mg/dL. The mean serum potassium level was 3.8± 0.3 mg/dL and the mean serum sodium level was 140.4± 3.4 mg/dL. In other words, patients with rhabdomyolysis did not have hyperkalemia, but their sodium levels were in the high range of normal. In our study, 5 of the 76 patients passed away due to renal insufficiency (6.5%), all of whom were male.

In the study of Taheri et al. [12], which tracked changing serum parameters in patients after taking opium and heroin, fasting blood glucose and potassium levels decreased significantly. Recent case studies of methadone abuse, morphine infusion, and the use of tramadol in different parts of the world have shown hyperglycemia (490.0 mg/dL), hyperkalemia (8.1 mmol/L), hypoglycemia, and hyponatremia [16-18]. In the study of Karam et al. [19], opium increased serum potassium and glucose levels in addicts with non-insulin dependent diabetes mellitus.

In the same study, the effects of opium on glucose, potassium, and sodium levels in males and females were investigated. Sodium, potassium, and glucose levels were significantly increased in all mice compared to the control group [20]. In the study of Talaei et al. [7], myoglobinuria had a prevalence of 13%, corresponding to the lower incidence of rhabdomyolysis in that study. However, myoglobin is rapidly filtered out through the kidneys, and the urine sampling time can also alter the results of myoglobin in patients with a catheter.

According to the results of this study, as well as results obtained from similar studies, it seems that the onset of rhabdomyolysis and accompanying electrolyte disorders can occur as soon as the day of admission, and therefore preventive and therapeutic procedures, such as adequate hydration and moving the patients out of the bed as soon as possible, offer a promising opportunity to prevent serious complications due to electrolyte disorders and rhabdomyolysis, which is associated with acute renal failure and death.

Based on the results of this and previous studies, rhabdomyolysis is more commonly associated with poisoning in younger patients and can lead to serious and fatal complications, such as progressive renal failure. Therefore, due to the high incidence of mortality as a result of poisoning in young people, it is necessary to pay more attention to early diagnosis and treatment of these patients. Future studies are recommended to be conducted on the following topics: the prevalence of rhabdomyolysis in different centers and locations, the accurate determination of its prevalence and risk factors, efficacy of treatment, and the chemical and molecular evaluation of toxins. Overall, a better understanding of the relationship between toxic substances and the phenomenon of rhabdomyolysis is required.

## Figures and Tables

**Table 1.** Comparison of the characteristics of the patients with and without rhabdomyolysis

	With rhabdomyolysis	Without rhabdomyolysis	Total	p-value
Age (mean±SD, yr)	35.45±4.82	39.78±6.31	37.69±5.87	0.03
Sex				
Male	69 (90.8)	222 (79.9)	291(82.2)	0.02
Female	7 (9.2)	56 (20.1)	63 (17.8)	
Type of drug				
Heroin	4 (5.3)	0 (0.0)	4 (1.1)	0.02
Cocaine	2 (2.6)	3 (1.1)	5 (1.4)	
Opium	24 (31.6)	93 (33.4)	117 (33.0)	
Tramadol	21 (27.6)	106 (38.1)	127 (35.9)	
Methadone	25 (32.9)	76 (27.3)	101 (28.5)	

Values are presented as number (%). SD, standard deviation.

**Table 2.** Number of hospitalized patients with rhabdomyolysis in the intensive care unit (ICU) according to type of drug and age group in 2014

Age group^1^	Heroin	Cocaine	Opium	Tramadol	Methadone	Total
11-19	0	0	0	5	1	6
20-29	0	0	3	14	5	22
30-39	1	1	10	2	8	22
40-49	1	1	6	0	6	14
50-59	0	0	4	0	5	9
60-69	1	0	0	0	0	1
70-79	1	0	0	0	0	1
80-89	0	0	1	0	0	1
Rhabdomyolysis incidence^2^						
First day of hospitalization	2	1	14	17	18	52
Subsequent days of hospitalization	2	1	10	4	7	24

1 No patients were 10 years old or younger.

2 Rhabdomyolysis incidence based on the onset of poisoning treated at the ICU.

**Table 3.** Average laboratory variables in patients with rhabdomyolysis

	Minimum	Maximum	Average	SD
LDH (IU/L)	161.0	3,266.0	921.5	511.8
Urea (mg/dL)	2.0	7.2	3.8	1.0
K^+^ (mg/dL)	2.8	5.0	3.8	0.3
Na^+^ (mg/dL)	134.0	150.0	140.4	3.4
Ca^2+^ (mg/dL)	0.6	7.4	1.4	1.1
P (mg/dL)	9.0	207.0	40.8	35.5

LDH, lactate dehydrogenase; K, potassium; Na, sodium; Ca, calcium; P, phosphorus; SD, standard deviation.
